# Integration of azithromycin mass administration to 1–11-month-old children into an existing health platform to reduce child mortality: a cluster-randomised trial in Burkina Faso

**DOI:** 10.1136/bmjgh-2025-021336

**Published:** 2026-01-08

**Authors:** Fanny Yago-Wienne, Djeinam Toure, Georges Dimithé, Regina Khassanova, Souleymane Sidibé, Pouorinibé Noel Some, Issouf Bamba, Valérie Zombré, Ali Sie, Mamadou Bountogo, Boubacar Coulibaly, Mamadou Ouattara, Brittany Peterson, Benjamin Arnold, Thomas M Lietman, Elodie Lebas, Kieran S O’Brien

**Affiliations:** 1 Helen Keller International, Ouagadougou, Burkina Faso; 2 Hellen Kelle Intl, Dakar, Senegal; 3 Ministère de la santé, Ouagadougou, Burkina Faso, Ouagadougou, Burkina Faso; 4 Centre de Recherche en Sante de Nouna, Nouna, Burkina Faso; 5 Francis I. Proctor Foundation, University of California San Francisco, San Francisco, California, USA; 6 Department of Epidemiology and Biostatistics, University of California San Francisco, San Francisco, California, USA; 7 Department of Opthalmology, University of California San Francisco, San Francisco, California, USA; 8 Institute for Global Health Sciences, University of California, San Francisco, San Francisco, CA, USA

**Keywords:** Africa, Global Health, Child health

## Abstract

**Background:**

Azithromycin mass drug administration (MDA) to ages 1–59 months can reduce childhood mortality; however, more evidence is needed to support targeting a narrower age range of 1–11 months. This trial assesses the efficacy of azithromycin MDA to 1–11-month-old children in reducing mortality in a real-world setting with integration of vitamin A delivery within the established Child Health Days platform in Burkina Faso.

**Methods:**

Mortalite Infantile Reduite par l’Administration de Masse de l’Azithromycine is a double-masked, cluster-randomised, placebo-controlled trial in Child Health Days communities in Burkina Faso. Primary healthcare centre catchment areas (Centres de Santé et de Promotion Sociale (CSPS)) were randomised 2:1 to deliver biannual azithromycin or placebo for 1–11-month-old children. Birth history data at the study endpoint were used to calculate mortality rates and compare between groups.

**Findings:**

From September 2021 to January 2024, 201 709 children in 303 CSPS received azithromycin and 100 959 children in 158 CSPS received placebo, with an overall treatment coverage of 85%. Mortality rates were 2.6 (95% CI 2.1 to 3.1) deaths per 1000 person-years in the azithromycin arm and 2.5 (95% CI 1.8 to 3.2) per 1000 person-years in the placebo arm. There was no significant difference in the mortality rates by arm (incidence rate ratio: 1.04; 95% CI 0.75 to 1.46; p value 0.80). There were 16 non-serious adverse events and no serious adverse events recorded during the trial.

**Interpretation:**

This trial demonstrates that azithromycin MDA for child survival can be scaled up and integrated into existing child health programmes but was unable to demonstrate an effect of azithromycin distribution on infant mortality. These findings indicate future policy decisions should consider treatment delivery to the larger age group of children up to 5 years old.

**Funding:**

The trial was funded by the Bill and Melinda Gates Foundation (INV-005395).

**Trial registration number:**

NCT04716712.

**WHAT IS ALREADY KNOWN ON THIS TOPIC**

- Several cluster-randomised trials in West Africa have shown a 14–18% decrease in childhood mortality with azithromycin mass drug administration (MDA) when targeted to 1–59-month-old children. WHO published guidelines to restrict distribution of treatment to 1–11-month-old children, although this more limited MDA had not yet been tested.

**WHAT THIS STUDY ADDS**

- This study evaluates whether targeting azithromycin MDA to 1–11-month-old children alone reduces mortality.

**HOW THIS STUDY MIGHT AFFECT RESEARCH, PRACTICE OR POLICY**

- This study adds to a growing body of evidence that restricting azithromycin MDA to 1–11-month-old children is not effective in reducing mortality in that age group, suggesting that the WHO guidelines should be revisited.

## Introduction

 Sub-Saharan Africa has the highest rate of child mortality in the world, with under-5 mortality rates of over 100 deaths per 1000 live births in some countries.^1^,^3^ The Sustainable Development Goals aim to reduce under-5 mortality to 25 per 1000 live births by 2030.^4^ Azithromycin mass drug administration (MDA) has been shown to reduce childhood mortality in several randomised controlled trials.^5^,^7^ WHO currently suggests consideration of the use of azithromycin MDA in high-mortality settings, although with restriction of distribution to 1–11-month-old children out of concern for antimicrobial resistance. Targeting this more limited age group alone had not yet been tested at the time the guidelines were released.^8^

As eligible countries in West Africa consider implementation of this intervention, there is interest in demonstrating that azithromycin MDA can be scaled up and integrated into standing programmes.^9^ The Child Health Days (CHD) platform used in Burkina Faso is one existing platform well situated for integration. CHD includes biannual community-based vitamin A supplementation, deworming and malnutrition screening targeting 6–59-month-old children, presenting substantial overlap with azithromycin MDA for child survival.^5^,^710 11^

The *Mortalite Infantile Reduite par l’Administration de Masse de l’Azithromycine* (MIRAMA) cluster-randomised, placebo-controlled trial compares the efficacy of mass distribution of azithromycin to 1–11-month-old children versus placebo in a real-world setting with integration into the CHD platform in Burkina Faso. MIRAMA aimed to both test the efficacy of limiting azithromycin MDA to 1–11-month-old children and demonstrate programme integration at a large scale. Given the community-based nature of the intervention and platform as well as the potential for herd effects, a cluster-randomised design was used.

## Methods

### Study design

MIRAMA was a double-masked, placebo-controlled, cluster-randomised trial in Burkina Faso. The trial compared all-cause mortality after 2 years in Centres de Santé et de Promotion Sociale (CSPS) catchment areas randomised to biannual (every 6 months) azithromycin to 1–11-month-old children or biannual placebo to 1–11-month-old children in settings receiving CHDs. CSPSs are primary healthcare centres with catchment areas of approximately five communities. CSPSs from three regions were included in the trial, based on having among the highest under-5 mortality rates and being accessible for data collection teams: Sud-Ouest, Centre-Est and Hauts-Bassins.^12^ The trial was registered at ClinicalTrials.gov (NCT04716712).

### Patient and public involvement

Neither patients nor the public were involved in the design of this study. However, the Burkina Faso Ministry of Health contributed to the study’s design. Additionally, the Burkina Faso Ministry of Health, CSPS leaders, community health workers and community leaders were engaged in the recruitment, implementation and dissemination phases of the study.

### Participants

The study took place from September 2021 to January 2024 in three regions of Burkina Faso: Sud-Ouest, Centre-Est and Hauts-Bassins. Randomisation occurred at the CSPS level, and data collection occurred at the village level within each CSPS. A village was eligible if it was in one of the three regions. Villages that were inaccessible or unsafe for the study team were excluded from census data collection; however, CHD and drug distribution activities still occurred. CHD and drug distribution activities were conducted by health teams that live in the areas being treated, whereas the data collection teams must come in from the outside. In inaccessible areas, people are not allowed to travel in from the outside but are able to continue other activities from within the communities. All children were eligible if they were 1–11 months old (30–364 days old), lived in a community participating in the study and had verbal consent from a guardian. Children were excluded if they had a known allergy to macrolides or were severely ill at the time of treatment distribution.

### Randomisation and masking

The trial randomised treatment in a 2:1 allocation favouring azithromycin. The allocation sequence was prepared by an unmasked biostatistician at the University of California, San Francisco, and linked to masked treatment bottles prepared by Pfizer. The placebo was indistinguishable from the active treatment, azithromycin, in appearance, smell and packaging. Treatment bottles were labelled with one of six letters, four for azithromycin and two for placebo to aid masking. All participants, study personnel, investigators and outcome assessors were masked to allocation until the primary analysis was completed. The primary analysis was done in a masked fashion using a re-randomised allocation sequence until the analysis was completed.

### Procedures

Data on mortality were collected in two ways throughout the study.^13^ First, a population-based census occurred at baseline and before every treatment distribution biannually. Trained census workers collected information regarding the eligible population, including demographic characteristics and vital status. Demographic data for heads of household, caregivers and children under 1 year of age were recorded. Vital status (alive, dead, moved or unknown) of included children was collected at each follow-up visit. New children and households were added at each census visit. Data were collected electronically using a mobile application (CommCare, Dimagi, Cambridge, Massachusetts, USA). Numbers of children treated were collected by the Ministry of Health during implementation, and eligible population denominators were estimated by the Ministry of Health using the 2019 census to calculate treatment coverage. Starting at the second census, verbal autopsy was carried out for all deceased children identified during previous censuses using the WHO 2016 VA questionnaire.^14^ Verbal autopsy interviews were conducted by trained personnel within 3 months of the census that captured the death and included questions regarding symptoms, medical history and circumstances before death to assign a probable cause of death.

After a masked interim analysis found lower-than-anticipated mortality rates from the census, the study team decided to include birth history data collection as the primary endpoint to allow for all deaths during the study period to be captured at a single time point in case they had been missed during the census. The birth history data collection used methods similar to those used in a related trial in Niger, as well as by the Demographic and Health Surveys programme.^15 16^ The birth history collected data from all 15–52-year-old women in all eligible households. The women were asked about all live births they had in the 3 years prior to the survey. Data on date of birth, current vital status and date of death were captured for each live birth. Data were collected using a similar mobile application as the census data collection (CommCare).

Treatment for this study was administered during routine biannual CHDs. CHDs are implemented by the Ministry of Health with support from Helen Keller International in both rural and urban areas. Established community health workers distribute the intervention in rural communities, and temporary community distributors paid for by the campaign do so in urban areas. The CHDs consist of door-to-door distribution of vitamin A along with screening for acute malnutrition to 6–59-month-old children and deworming with albendazole or mebendazole for 12–59-month-old children. MIRAMA leveraged this platform to implement the biannual administration of azithromycin to 1–11-month-old children. In order to incorporate MDA of azithromycin, some logistical additions were made to the programme such as training pertaining to distribution of azithromycin, activities for sensitisation of communities, advocacy meetings for administrative authorities and introduction of transport and logistics for azithromycin drug supply. Azithromycin and placebo were administered as a dose of oral suspension determined by age for children under 6 months and height for children 6 months and older, reconstituted with water in the field.^17^ This trial used age and height-based dosing for azithromycin rather than weight-based dosing due to the ease of determining dose and the logistical complexity of using weighing scales when delivering azithromycin at such a large scale. Age and height dosing has been found to result in high accuracy.^17^ A second dose was administered if the child vomited directly after taking the drug. Caregivers were encouraged to report any adverse events occurring within 48 hours of treatment and were given up to 28 days to report to a community health worker or facility health worker via the national pharmacological monitoring system and communicated to the study team.

### Outcomes

The primary outcome for this trial was the mortality rate among 1–11-month-old children as measured by deaths per 1000 person-years at risk at the CSPS level. Using the birth history approach, deaths were counted as those that occurred within 6 months of a community’s MDA among children who were eligible for treatment. Person-time at risk was calculated as the time a child was alive and eligible for treatment while living in the study area in that 6-month period.

The secondary outcome was the mortality rate measured using the biannual census. The mortality was measured as deaths per 1000 person-years at risk at the CSPS level. A child was counted as a death if they were present on one census and absent on the subsequent census due to death. Person-time at risk was calculated as time alive and eligible for treatment while living in the study area between each inter-census interval. Children who died contributed half of the person-time in their last inter-census interval, and children who moved or had an unknown status contributed no person-time to the analysis. An exploratory outcome included cause-specific mortality as ascertained by verbal autopsy.

### Statistical analysis

Based on data from the first census, the trial planned to treat at least 92 000 children aged 1–11 months at each treatment distribution. With each child being followed every 6 months and assuming an estimated loss to follow-up rate of 10%, this led to an estimated 165 600 person-years of observation within 516 CSPSs, or about 327 person-years of observation per cluster. The mortality rate calculation was informed by two mortality trials with expected similar mortality incidence rates, CHAT in Burkina Faso and MORDOR in Malawi.^5 6^ Based on these estimates, the assumed mortality rate in the control arm was 10 per 1000 person-years and the coefficient of variation for CSPS-level mortality was 0.33. Given a treatment allocation of 2:1 and two-sided alpha of 0.05, we estimated that the study would have 80% power to detect a 16% relative reduction in the mortality rate among 1–11-month-old children using a standard sample size equation for comparison of incidence rates in a cluster-randomised trial, with 302 cluster-level df and a design effect of 1.38.^18^ Further assuming 10% loss-to-follow-up of CSPSs, the trial’s detectable effect was a 17% relative reduction in mortality.

The prespecified primary analysis was a Poisson regression with robust SEs clustered at the CSPS level to estimate the incidence rate ratio (IRR) comparing mortality in the azithromycin arm to the placebo arm. The model included the number of deaths per cluster as the outcome with treatment group as a predictor and the cluster-level person-time at risk as an offset. The incidence rate difference (IRD) was determined by calculating the difference in mortality rates by arm, and the 95% CI was estimated using a non-parametric bootstrap that resampled CSPSs with replacement (10 000 replicates). Hypothesis testing was two-sided with alpha of 0.05 for all analyses. Statistical significance was assessed, with permutation p values determined with Monte Carlo permutation testing with 10 000 replicates. Both analyses of census and birth history methods of enumerating deaths and person-time were conducted similarly. Mortality rates measured by each method were compared using Pearson’s correlation. Given that mortality rates were lower than anticipated, a probabilistic bias analysis was conducted using published estimates of the sensitivity of birth history to correctly classify deaths.^15^ Corrected IRDs were simulated across 10 000 iterations, and within each iteration the number of deaths by randomisation unit was adjusted following a beta distribution based on the prior observed sensitivity. Monte Carlo-simulated random error based on the variance of the IRD was added, allowing generation of a bias-adjusted and error-adjusted 95% uncertainty interval. A secondary analysis of the infant mortality rate was done by estimating the cumulative probability of mortality in each treatment group using a synthetic cohort approach, consistent with the Demographic and Health Surveys, using age groups 0, 1–2, 3–5 and 6–11 completed months.^19^ Prespecified subgroup analyses compare the IRRs by arm among the following subgroups: age group (1–5 months vs 6–11 months), sex and region. A non-prespecified subgroup analysis by CSPS type (urban, rural and mixed) is also included.

An exploratory analysis compared cause-specific mortality outcomes between arms. Verbal autopsy data were analysed using the InterVA5 package in R which determined the primary cause of death.^20^ This package uses an algorithm which matches each child’s reported symptoms before death to a profile for each of the most common causes of death in low-resource settings. Only causes with >20% likelihood according to the InterVA5 algorithm were used in the analysis. Further, causes of death had to have been represented by >5 children overall and at least 1 child per arm. IRRs comparing the two treatment arms for each cause of death were calculated using Poisson regression using the same methods as the primary analysis, with number of deaths for each specific cause as the outcome in separate models. P values were adjusted for multiple comparisons using a Bonferroni correction.

## Results

**Figure 1:**
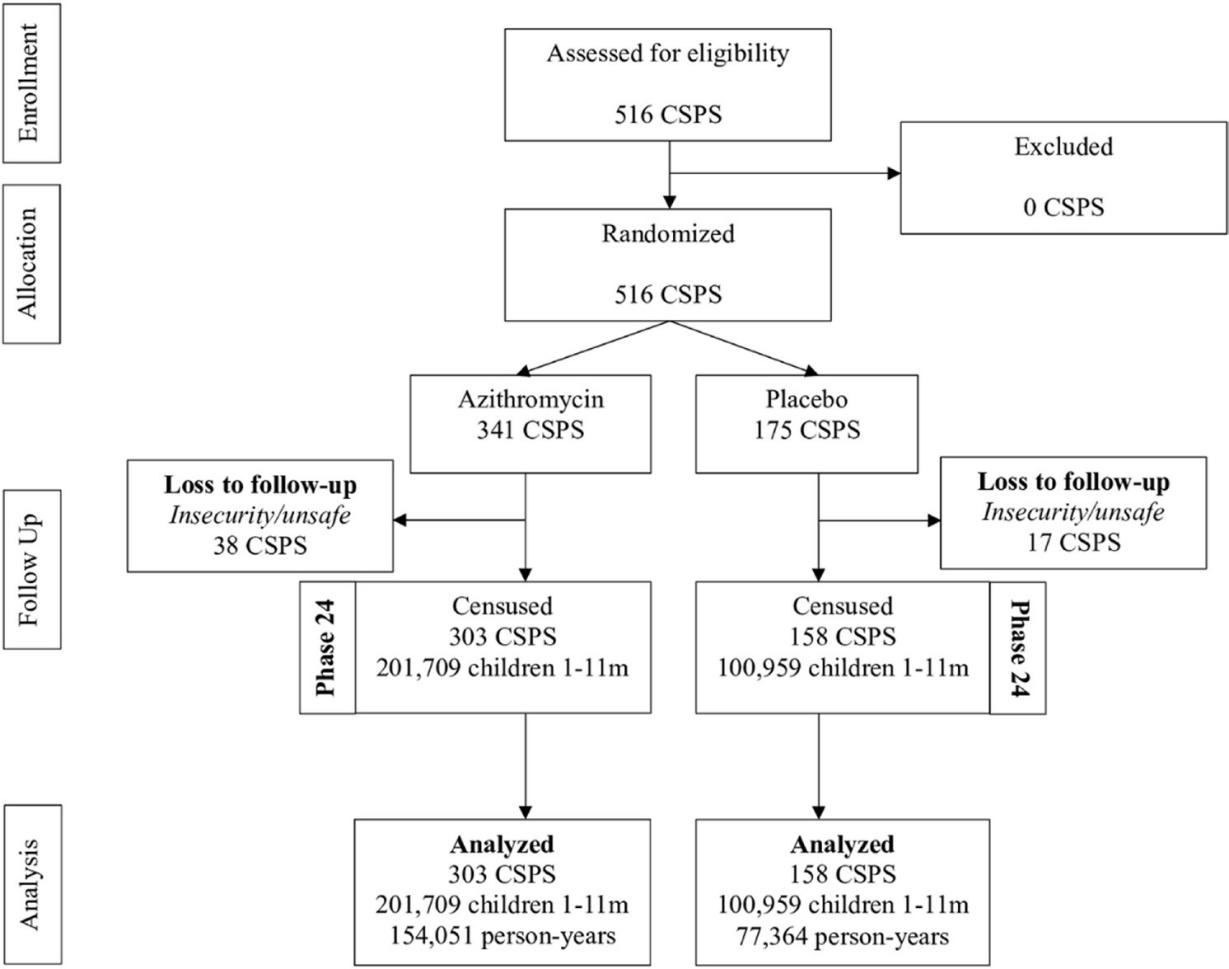
Participant flow based on birth history data collection. CSPS, Centres de Santé et de Promotion Sociale.

There were 516 clusters assessed for eligibility into the trial. All clusters assessed for eligibility were randomised, with 341 randomised to azithromycin and 175 to placebo (figure 1). Birth history data collection occurred during the last census of the study, and 55 clusters were not able to be reached due to insecurity (38 in the azithromycin arm and 17 in the placebo arm). For census data collection, a total of 12 clusters were lost to follow-up and not included in the analysis due to insecurity, 6 in the azithromycin arm and 6 in the placebo arm (online supplemental figure 1). Baseline characteristics of children censused at the first round of data collection within CSPS contributing person-time to the primary analysis were similar between groups (table 1). Comparison of baseline characteristics for clusters included in census data collection and clusters included in birth history data collection shows that the two groups of clusters are similar (online supplemental table 1). Treatment coverage in the first round was 58.4% and in all other rounds exceeded 90% (online supplemental table 2).^1^

**Table 1:** Community-level baseline characteristics of children at baseline census visit among CSPS contributing person-time to primary analysis

Characteristics	Azithromycin N=332	Placebo N=168
Children (N)	64 647	30 699
Children per CSPS		
Mean (SD)	178.54 (159.9)	166.85 (135.23)
Percent female (N, %)	31 733, 49.1%	15 071, 49.1%
Age group (N, %)		
1–5 months	28 652, 44.3%	13 536, 44.1%
6–11 months	30 623, 47.4%	14 495, 47.2%

CSPS, Centres de Santé et de Promotion Sociale.

**Figure 2:**
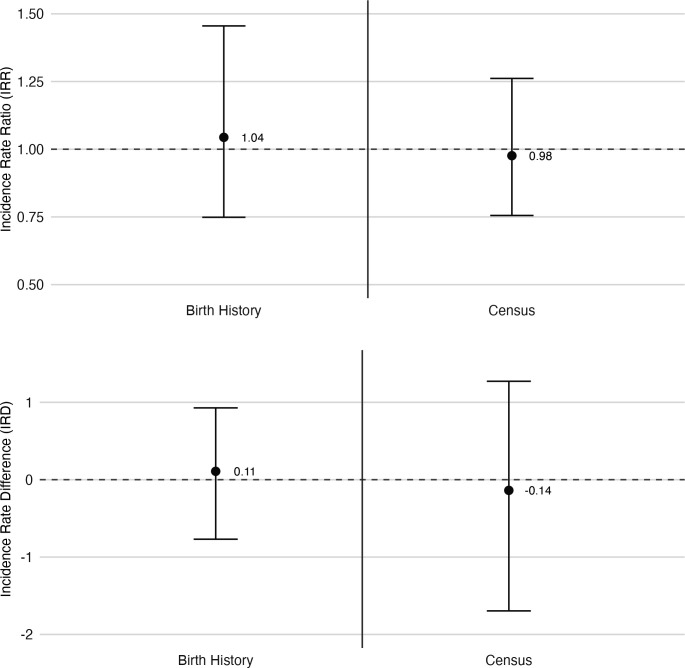
Incidence rate ratios and incidence rate differences comparing 1–11-month mortality in azithromycin and placebo clusters.

Overall, 302 668 children and 231 415 person-years contributed to the primary outcome (table 2). The azithromycin arm recorded 397 deaths and 154 051 person-years while the placebo arm recorded 191 deaths and 77 364 person-years. Mortality rates were 2.6 (95% CI 2.1 to 3.1) deaths per 1000 person-years in the azithromycin arm and 2.5 (95% CI 1.8 to 3.2) per 1000 person-years in the placebo arm. There was no significant difference in the mortality rates by arm (IRR 1.04; 95% CI 0.75 to 1.46; p value 0.80) (figure 2). Results were similar after accounting for potential outcome misclassification with the bias analysis (IRD 0.13, 95% uncertainty interval: −0.34 to 0.62). Subgroup analyses found no differences in mortality by age group, sex, region or CSPS type (online supplemental table 3).

**Table 2:** Number of children, deaths, person-years and mortality rates in each arm for both data collection methods

Characteristics	Birth history		Census	
	Azithromycin N=201 709	Placebo N=100 959	Azithromycin N=167 018	Placebo N=80 878
Deaths (n)	397	191	691	345
Person-years (n)	154 051	77 364	122 519	59 710
Mortality rate per 1000 person-years (95% CI)	2.6 (2.1 to 3.1)	2.5 (1.8 to 3.2)	5.6 (4.9 to 6.4)	5.8 (4.7 to 7.2)

The secondary outcome analysis using the census data collection included 247 896 children and 182 229 person-years (table 2). There were 691 deaths and 122 519 person-years in the azithromycin arm, and 345 deaths and 59 710 person-years in the placebo arm. Mortality rates were 5.6 (95% CI 4.9 to 6.4) in the azithromycin arm and 5.8 (95% CI 4.7 to 7.2) in the placebo arm. Again, no significant difference was found in the mortality rates (IRR 0.98; 95% CI 0.76 to 1.26; p value 0.85) (figure 2). Pearson’s correlation of mortality rates from both birth history and census data collection methods was 0.45 (online supplemental figure 2).

The secondary analysis estimating cumulative probabilities of mortality using a synthetic cohort approach included 97 205 live births in the placebo group and 193 248 live births in the azithromycin group. The placebo group included 247 deaths, and the azithromycin group included 496 deaths (online supplemental table 4). Mortality was 3.3 deaths per 1000 live births (95% CI 2.7 to 3.9) in azithromycin communities and 3.1 (95% CI 2.3 to 3.9) in placebo communities. No significant difference was found in mortality (IRR 1.05; 95% CI 0.77 to 1.45; p value 0.76).

**Figure 3:**
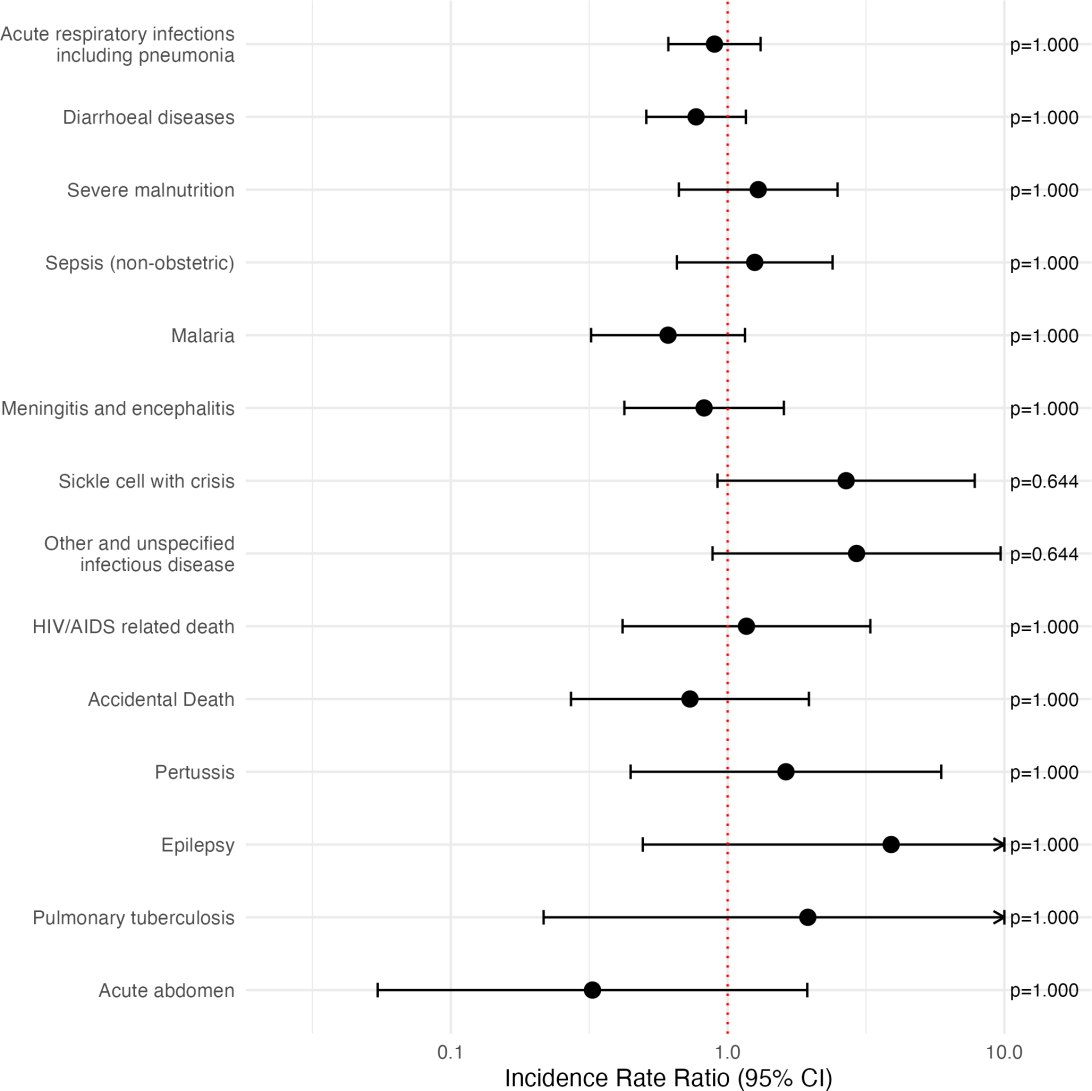
Incidence rate ratios of cause-specific deaths from verbal autopsies in order from most common to least common with p values adjusted using a Bonferroni correction.

1265 verbal autopsies were conducted throughout the study, accounting for 78% of the total 1624 deaths that occurred. 598 of these verbal autopsies were not included as their deaths did not contribute to the primary analysis due to being out of age range and 15 verbal autopsies were not analysed by the InterVA5 package due to incomplete data. The analysis included 436 verbal autopsies in the azithromycin arm and 216 in the placebo arm. The most common causes of death were acute respiratory infection, including pneumonia (24.4%), diarrhoeal disease (22.5%) and severe malnutrition (9.5%). There were no causes of death with significant differences between arms (figure 3, online supplemental table 5).

There were 16 non-serious adverse events and no serious adverse events recorded during the trial. 12 were in the azithromycin arm and 4 in the placebo arm, and all were related to diarrhoea or vomiting. No adverse events were attributed to treatment.

## Discussion

The MIRAMA trial compared the efficacy of mass distribution of azithromycin to 1–11-month-old children versus placebo integrated into a standing vitamin A supplementation programme in Burkina Faso at a large scale. No significant difference in mortality rates was found in communities receiving azithromycin and placebo; however, mortality rates were lower than those anticipated with the sample size calculation. This lack of a significant difference was seen with both the birth history census approaches to collecting mortality data. However, the successful incorporation into the CHD platform with high treatment coverage demonstrates a promising strategy for integration of child health programmes at a large scale.

The mortality rates found in this study were lower than anticipated, reducing statistical power to detect a difference between arms.^5^,^7^ Assuming the mortality rates seen in the trial, we had 10% power to detect the original target effect size of 16%. A few recent child mortality studies in Burkina Faso have also found lower mortality rates than expected based on prior national surveys.^6 21 22^ In addition, Burkina Faso has been consistently reporting lower-than-anticipated mortality rates which have been attributed to intensive efforts targeting child survival in the past few years, including providing free care for children and bolstering existing programmes. This trial also included urban areas, while other similar trials finding higher mortality rates in infants only included rural areas.^5^,^7^ Urban areas typically experience lower mortality rates and have greater access to healthcare, and their inclusion in this trial could have lowered the overall rates, ultimately decreasing power to detect differences. Accounting for potential outcome misclassification did not substantially alter the estimated effect, and the wide uncertainty interval suggests that the true IRD may be small or null. The findings emphasise the likelihood that mortality rates in this setting are genuinely low, consistent with recent trends and national efforts to improve child survival.

The study team attempted to address the potential for underreporting of deaths by including a second form of mortality data collection, though mortality rates in this study may still have been underestimated. The birth history method asked caregivers to recall all live births in the past 3 years, while the census data collection method only required a recall period of 6 months. It is possible that poor recall over longer periods of time could lead to the inability to capture all deaths and live births seen, hence the lower mortality in the birth history approach relative to the census.^23 24^ Also, previous similar trials offered treatment at the same time as the census data collection; however, in MIRAMA, census and treatment were done separately and no treatment was offered during the door-to-door census. The lack of requirement for a census worker to treat a child could have led to under-reporting of mortality in both data collection methods. While these factors may have led to underestimation of the mortality rates, given the placebo-controlled design, we do not expect this bias to be differential by arm. In addition, we found a reasonable correlation between mortality rates estimated by the two methods.

Two recent trials compared the distribution of azithromycin versus placebo in younger children. The CHATON trial in Burkina Faso restricted distribution to 5–12-week-old children and was unable to find a reduction of mortality by 6 months of age in children receiving azithromycin.^22^ The LAKANA trial in Mali compared biannual and quarterly azithromycin treatment to placebo among 1–11-month-old infants and found no reductions in infant or child mortality among either arm.^25^ Most recently, the AVENIR trial investigated the effects of azithromycin distribution on child mortality by separately comparing azithromycin MDA to two age groups against placebo: 1–59-month-old children and 1–11-month-old children.^7^ The findings revealed a 14% reduction in child mortality in communities where azithromycin was administered to 1–59-month-old children compared with the placebo group.^7^ However, there was no significant reduction in child mortality observed in communities where azithromycin was given to 1–11-month-old children compared with placebo.^7^ Further, the AVENIR results suggested the presence of an indirect effect of treating older children on mortality in 1–11-month-old children, with a 17% reduction in 1–11-month mortality if 12–59-month-old children were also treated.^7^ Older children are likely driving transmission since they have higher exposure than the youngest children, thus treating older children would reduce transmission and result in this indirect benefit to younger children. A secondary analysis of this trial found results consistent with a spillover effect of treatment to 1–11-month-old children when 12–59-month-old siblings were treated in the household.^26^ Treating a broader age group than 1–11-month-old children alone may be required to ensure widespread benefits. These findings align with the results presented here and emphasise that the benefits of biannual distribution of azithromycin on child mortality are not identified when delivering treatment to only <1-year-old infants. Because prior studies have found a mortality benefit with treating a larger age group of 1–59-month-old children, the inclusion of broader age groups should be explored. Other studies of treatment with 1–59-month-old children have suggested heterogeneity in the effect by baseline mortality rate, but this trial was not powered to examine effect modification by subgroups.

Biannual administration of azithromycin has been shown to increase antimicrobial resistance in similar settings.^27^,^30^ Due to this threat, the WHO published guidelines targeting azithromycin to only 1–11-month-old children.^8^ While the rationale for limiting drug distribution was to reduce AMR, this limiting would also reduce the number of deaths averted overall.^31^ Limiting the age group eligible for MDA has not proven effective in any trial published to date.^7^

This study has several limitations. First, as child mortality is a rare event, a large sample size is needed to be able to detect a significant difference. In this analysis, we found lower-than-expected mortality rates which led to reduced power to detect small differences. Second, other child mortality trials in West Africa have used one team to both deliver drug and conduct censuses; however, in MIRAMA, the treatment data collection was done by the Ministry of Health, and the census and birth history data collections were contracted to an outside agency. Children may have been missed during census data collection, which could have led to the lower-than-expected mortality rates. Another reason for this could be the inclusion of urban areas in which it is more difficult to find and census all households. However, we anticipate that any bias introduced by missing children in the census would not differ between arms. Further, the correlation between mortality rates calculated using census data and birth history was 0.45, which is quite reasonable for a rare outcome like deaths and indicates the two methods are comparable. MIRAMA also excluded seriously ill children from treatment, whereas MORDOR, CHAT and AVENIR included all children, regardless of presentation with illness. A portion of the effectiveness of azithromycin could be due to treating these seriously ill children, which was not captured in MIRAMA. Another limitation is that with the introduction of the birth history data collection in the final round, there were 55 clusters lost to follow-up due to insecurity in those areas. Not only could data collection teams not enter these areas, but it is also possible that in clusters they were able to enter, they may have missed households due to displacement or differing levels of insecurity within one cluster. However, baseline characteristics between the clusters included in the birth history data collection and the census were similar. We have no reason to believe that the intervention was associated with the loss to follow-up and do not expect selection bias.

The MIRAMA trial demonstrated that azithromycin MDA for child survival can be scaled up and integrated into existing child health programmes. The trial was unable to demonstrate an effect of azithromycin distribution on infant mortality. Follow-up studies will describe the implementation and associated costs of the integration, in addition to the impact of this intervention on antibiotic resistance. When considered together with the results of other recent trials, these findings suggest the importance of treating children up to 5 years old in MDA programmes for child survival.

## Supplementary material

10.1136/bmjgh-2025-021336 online supplemental file 1

10.1136/bmjgh-2025-021336 online supplemental file 2

## Data Availability

Data are available in a public, open access repository.
