# Supplementary material for: Integration of azithromycin mass administration to 1–11-month-old children into an existing health platform to reduce child mortality: a cluster-randomised trial in Burkina Faso
Source: BMJ Glob Health. 2026 Jan 8;11(1):e021336. doi: 10.1136/bmjgh-2025-021336 (PMC13059839; doi:10.1136/bmjgh-2025-021336)
Supplement: online supplemental file 1 [file bmjgh-11-1-s001.docx]

**Supplemental Figure 1.** Participant flow based on census data collection


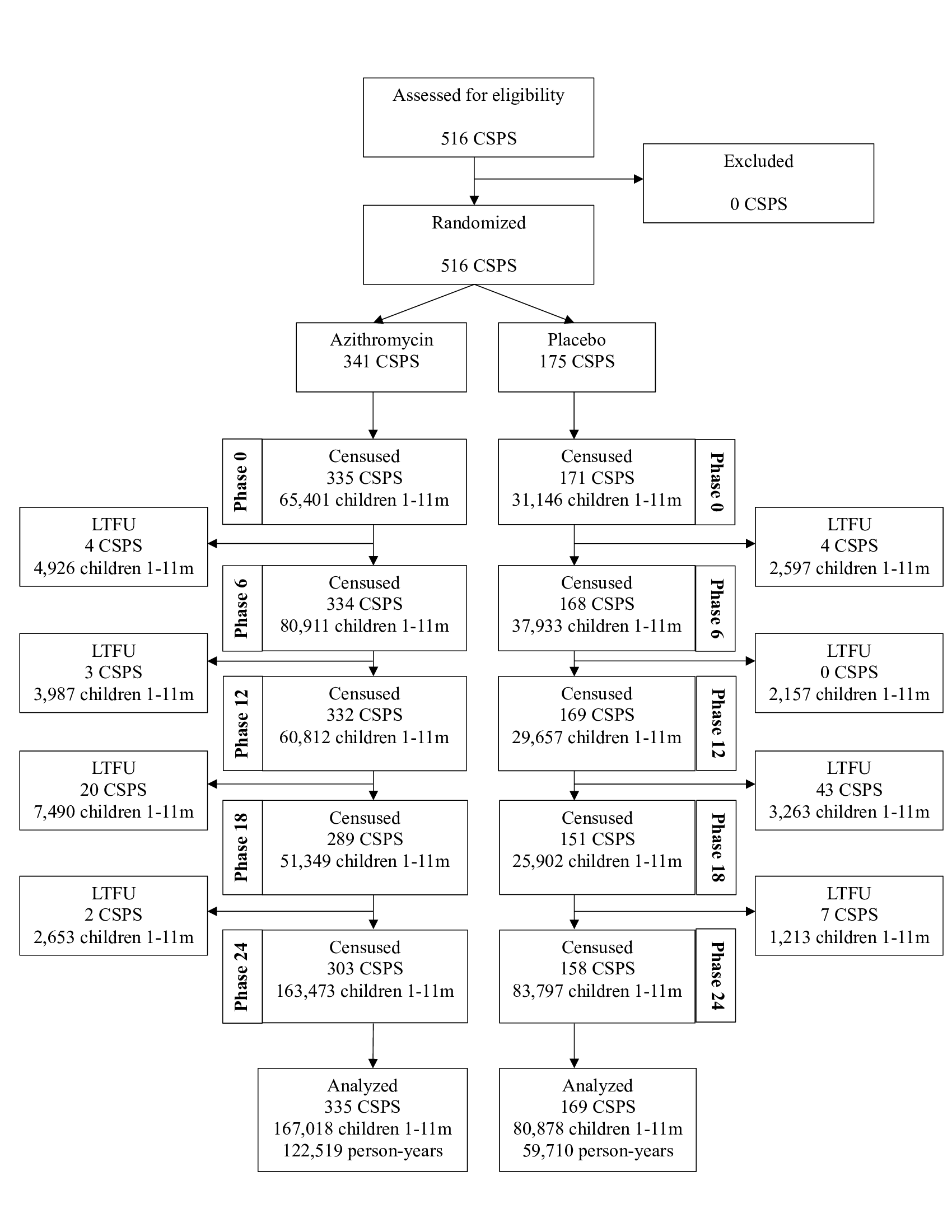


**Supplemental Figure 2.** Correlation among 1-11 month mortality rates at the CSPS level measured using census and birth history data collection methods


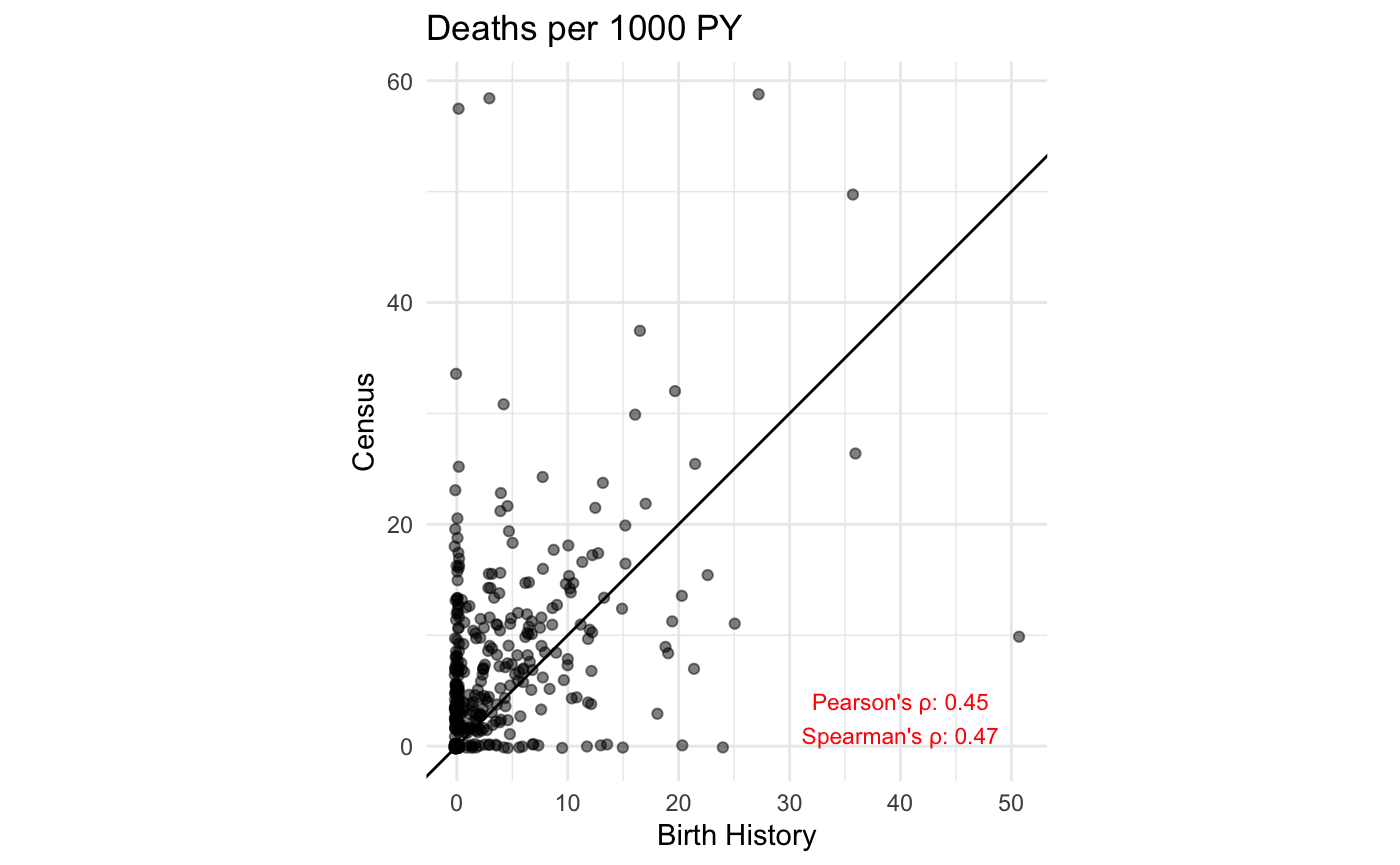


* PY = person-years

**Supplemental Table 1.** Characteristics of children at the baseline census visit comparing clusters included in birth history data collection and clusters included in census data collection.

| **Characteristic** | Birth History* | | Census | |
| --- | --- | --- | --- | --- |
|  | Azithromycin  N = 300 | Placebo  N = 157 | Azithromycin  N = 332 | Placebo  N = 168 |
| **Children (N)** | 57,324 | 29,015 | 64,647 | 30,699 |
| **Children per CSPS**  **Mean (SD)** | 174.79 (151.31) | 168.62 (138.61) | 178.54  (159.9) | 166.85 (135.23) |
| **Percent female (N, %)** | 28,083, 49.0% | 14,303,  49.3% | 31,733, 49.1% | 15,071, 49.1% |
| **Age Group (N, %)** |  |  |  |  |
| **1-5 months** | 25,674, 44.8% | 12,805, 44.1% | 28,652, 44.3% | 13,536, 44.1% |
| **6-11 months** | 26,764, 46.7% | 13,668, 47.1% | 30,623, 47.4% | 14,495, 47.2% |

* Baseline characteristics for birth history are calculated at census baseline only including CSPS that were visited for birth history collection

**Supplemental Table 2.** Treatment coverage by region and round using administrative coverage captured by the Ministry of Health

| **Round** | **Region** | **Treated** | **Eligible** | **Coverage** |
| --- | --- | --- | --- | --- |
| 1 | Centre-Est | 40,072 | 61,700 | 65.0% |
|  | Hauts-Bassins | 35,590 | 83,599 | 42.6% |
|  | Sud-Ouest | 28,794 | 33,437 | 86.1% |
|  | Overall | 104,456 | 178,736 | 58.4% |
| 2 | Centre-Est | 55,104 | 61,538 | 89.5% |
|  | Hauts-Bassins | 63,603 | 71,179 | 89.4% |
|  | Sud-Ouest | 32,080 | 31,998 | 100.3% |
|  | Overall | 150,787 | 164,715 | 91.5% |
| 3 | Centre-Est | 56,016 | 61,538 | 91.0% |
|  | Hauts-Bassins | 74,655 | 71,179 | 104.9% |
|  | Sud-Ouest | 32,700 | 31,752 | 103.0% |
|  | Overall | 163,371 | 164,469 | 99.3% |
| 4 | Centre-Est | 54,228 | 62,362 | 87.0% |
|  | Hauts-Bassins | 70,754 | 73,219 | 96.6% |
|  | Sud-Ouest | 31,147 | 32,504 | 95.8% |
|  | Overall | 156,129 | 168,085 | 92.9% |
| Overall | Centre-Est | 205,420 | 247,138 | 83.1% |
|  | Hauts-Bassins | 244,602 | 299,176 | 81.8% |
|  | Sud-Ouest | 124,721 | 129,691 | 96.2% |
|  | Overall | 574,743 | 676,005 | 85.0% |

* Total number of children censused over all rounds is 383,120

**Supplemental Table 3.** Number of live births, deaths, infant mortality rates, incidence rate ratio, and *P-*value for subgroup analyses using birth history data

| **Subgroup** | | **Azithromycin** | | | **Placebo** | | | **Azithromycin vs Placebo** | | **Interaction *P*-value** |
| --- | --- | --- | --- | --- | --- | --- | --- | --- | --- | --- |
|  |  | Deaths | Person-years | Infant Mortality Rate* (95% CI) | Deaths | Person-years | Infant Mortality Rate* (95% CI) | Incidence Rate Ratio (95% CI) | Permutation *P*-value |  |
| Age Group | 1-5 months | 177 | 62,794 | 2.8  (2.2 – 3.5) | 72 | 31,383 | 2.3  (1.7 – 3.0) | 1.23  (0.84 – 1.81) | 0.31 | Ref |
|  | 6-11 months | 196 | 80,335 | 2.4  (1.9 – 3.0) | 105 | 40,516 | 2.6  (1.8 – 3.6) | 0.94  (0.63 – 1.45) | 0.77 | 0.21 |
| Sex | Female | 199 | 71,141 | 2.8  (2.2 – 3.4) | 102 | 35,759 | 2.9  (2.0 – 3.9) | 0.98  (0.67 – 1.47) | 0.93 | Ref |
|  | Male | 198 | 82,910 | 2.4  (1.9 – 2.9) | 89 | 41,605 | 2.1  (1.5 – 2.8) | 1.12  (0.78 – 1.62) | 0.55 | 0.48 |
| Region | Centre-Est | 106 | 60,512 | 1.8  (1.3 – 2.2) | 56 | 33,794 | 1.7  (1.2 – 2.2) | 1.06  (0.72 – 1.60) | 0.79 | Ref |
|  | Sud-Ouest | 262 | 36,142 | 7.2  (6.0 – 8.6) | 120 | 15,489 | 7.7  (5.7 – 10.1) | 0.94  (0.67 – 1.33) | 0.69 | 0.64 |
|  | Hauts-Bassins | 29 | 57,398 | 0.5  (0.3 – 0.7) | 15 | 28,081 | 0.5  (0.3 – 0.9) | 0.95  (0.52 – 1.95) | 0.85 | 0.76 |
| CSPS Type | Rural | 313 | 82,508 | 3.8  (3.1 – 4.6) | 129 | 39,937 | 3.2  (2.4 – 4.2) | 1.17  (0.83 – 1.69) | 0.36 | Ref |
|  | Urban | 26 | 40,964 | 0.6  (0.5 – 0.8) | 11 | 14,812 | 0.7  (0.3 – 1.2) | 0.85  (0.48 – 1.99) | 0.60 | 0.38 |
|  | Mixed | 58 | 30,568 | 1.9  (1.3 – 2.6) | 51 | 22,451 | 2.3  (1.1 – 3.8) | 0.84  (0.42 – 1.88) | 0.64 | 0.39 |

* The infant mortality rate is the number of infant deaths per 1,000 person-years.

**Supplemental Table 4.** Number of live births, deaths, infant mortality rates, incidence rate ratio, and *P-*value for the analysis utilizing cumulative probabilities and a synthetic cohort approach.

| **Azithromycin** | | | **Placebo** | | | **Azithromycin vs Placebo** | |
| --- | --- | --- | --- | --- | --- | --- | --- |
| Live births | Deaths | Infant Mortality Rate*  (95% CI) | Live births | Deaths | Infant Mortality Rate* (95% CI) | Incidence Rate Ratio (95% CI) | Permutation *P*-value |
| 193,248 | 496 | 3.3  (2.7 – 3.9) | 97,205 | 247 | 3.1  (2.3 – 3.9) | 1.05  (0.77 – 1.45) | 0.76 |

* The infant mortality rate (infant deaths per 1,000 live births) is the probability of a child exposed in a specific period dying before reaching their first birthday.

**Supplemental Table 5.** Number of deaths by arm and incidence rate ratio for cause-specific deaths from verbal autopsy

| **Cause** | **Number of Deaths** | | **Incidence Rate Ratio (95% CI)** | **Permutation**  ***P*-value*** |
| --- | --- | --- | --- | --- |
|  | Azithromycin | Placebo |  |  |
| Acute respiratory infection including pneumonia | 103 | 56 | 0.90 (0.61 – 1.32) | 1.00 |
| Diarrhoeal diseases | 90 | 57 | 0.77 (0.51 – 1.16) | 1.00 |
| Severe malnutrition | 45 | 17 | 1.29 (0.67 – 2.50) | 1.00 |
| Sepsis (non-obstetric) | 36 | 14 | 1.25 (0.66 – 2.39) | 1.00 |
| Malaria | 25 | 20 | 0.61 (0.32 – 1.16) | 1.00 |
| Meningitis and encephalitis | 27 | 16 | 0.82 (0.42 – 1.60) | 1.00 |
| Sickle cell with crisis | 22 | 4 | 2.68 (0.92 – 7.81) | 0.64 |
| Other and unspecified infectious disease | 18 | 3 | 2.92 (0.88 – 9.69) | 0.64 |
| HIV/AIDS related death | 12 | 5 | 1.17 (0.42 – 3.28) | 1.00 |
| Accidental death** | 9 | 6 | 0.73 (0.27 – 1.97) | 1.00 |
| Pertussis | 10 | 3 | 1.62 (0.45 – 5.92) | 1.00 |
| Epilepsy | 8 | 1 | 3.90 (0.49 – 30.78 | 1.00 |
| Pulmonary tuberculosis | 4 | 1 | 1.95 (0.22 – 17.56) | 1.00 |
| Acute abdomen | 2 | 3 | 0.32 (0.05 – 1.94) | 1.00 |

*Adjusted using a Bonferroni correction

**Includes accidental fall, accidental exposure to smoke, fire & flame, road traffic accident, accidental drowning and submersion, other transport accident, and accidental poisoning & noxious substance
