# Supplementary material for: Integration of azithromycin mass administration to 1–11-month-old children into an existing health platform to reduce child mortality: a cluster-randomised trial in Burkina Faso
Source: BMJ Glob Health. 2026 Jan 8;11(1):e021336. doi: 10.1136/bmjgh-2025-021336 (PMC13059839; doi:10.1136/bmjgh-2025-021336)
Supplement: online supplemental file 2 [file bmjgh-11-1-s002.docx]

### BMJ Global Health Author Reflexivity Statement

Adapted from Morton, B., Vercueil, A., Masekela, R., Heinz, E., Reimer, L., Saleh, S., Kalinga, C., Seekles, M., Biccard, B., Chakaya, J., Abimbola, S., Obasi, A. and Oriyo, N. (2022), Consensus statement on measures to promote equitable authorship in the publication of research from international partnerships. Anaesthesia, 77: 264-276. <https://doi.org/10.1111/anae.15597>

| **Study conceptualisation** | |
| --- | --- |
| 1. How does this study address local research and policy priorities? | This trial assesses the efficacy of azithromycin MDA to children 1-11 months old in reducing mortality in a real-world setting with integration of vitamin A delivery within the established Child Health Days platform in Burkina Faso. Both integration into the existing platform and efficacy of the treatment are research and policy priorities in Burkina Faso. |
| 1. How were local researchers involved in study design? | The Burkina Faso Ministry of Health, Burkina-based staff from Helen Keller International, and the local Centre de Recherche en Sante de Nouna team contributed to the conception of the study's design. |
| **Research management** | |
| 1. How has funding been used to support the local research team(s)? | Only local research teams were hired to perform data collection. Teams were trained on electronic data collections, creation of electronic data collection platforms. |
| **Data acquisition and analysis** | |
| 1. How are research staff who conducted data collection acknowledged? | Those in charge of data collection are included as authors and those who collected data are acknowledged in the manuscript as part of the MIRAMA study group. |
| 1. How have members of the research partnership been provided with access to study data? | Data is stored in a public repository available for anyone to access. |
| 1. How were data used to develop analytical skills within the partnership? | Data access was given to Burkina Faso data managers to work with themselves and any questions they had were addressed by the UCSF data team. |
| **Data interpretation** | |
| 1. How have research partners collaborated in interpreting study data? | Research partners participated in virtual meetings to discuss data reports and interpretation of the study data. |
| **Drafting and revising for intellectual content** | |
| 1. How were research partners supported to develop writing skills? | All manuscripts were written in collaboration with local teams |
| 1. How will research products be shared to address local needs? | Published manuscript will be widely shared with the Ministry of Health in Burkina Faso and other stakeholders. |
| **Authorship** | |
| 1. How is the leadership, contribution and ownership of this work by LMIC researchers recognised within the authorship? | LMIC researchers are included first in the author list. |
| 1. How have early career researchers across the partnership been included within the authorship team? | Early career researchers were in charge or data collection, manuscript writing, and given authorship. |
| 1. How has gender balance been addressed within the authorship? | 7 authors are female and the first two authors and corresponding authors are female. |
| **Training** | |
| 1. How has the project contributed to training of LMIC researchers? | The project contributed to LMIC capacity by training local researchers in study design and protocol development, implementation of study procedures, data management, and laboratory methods. |
| **Infrastructure** | |
| 1. How has the project contributed to improvements in local infrastructure? | This project allowed the integration of this platform into established Child Health Days which is now being utilized for national roll out. |
| **Governance** | |
| 1. What safeguarding procedures were used to protect local study participants and researchers? | We obtained local and institutional ethics approval, used informed consent, maintained confidentiality, and trained staff in safety procedures to protect participants and researchers. |
